# Volumetric Analysis of the Hypothalamus in Huntington Disease Using 3T MRI: The IMAGE-HD Study

**DOI:** 10.1371/journal.pone.0117593

**Published:** 2015-02-06

**Authors:** Sanaz Gabery, Nellie Georgiou-Karistianis, Sofia Hult Lundh, Rachel Y. Cheong, Andrew Churchyard, Phyllis Chua, Julie C. Stout, Gary F. Egan, Deniz Kirik, Åsa Petersén

**Affiliations:** 1 Translational Neuroendocrine Research Unit, Department of Experimental Medical Science, Lund University, Lund, Sweden; 2 School of Psychological Sciences, Monash University, Clayton, Victoria, 3180, Australia; 3 Huntington’s Disease Unit, Bethlehem Hospital, Kooyong Rd, Caulfield, Victoria, 3162, Australia; 4 Department of Psychiatry, School of Clinical Sciences at Monash Health, Monash University, Clayton, Victoria, 3168, Australia; 5 Monash Biomedical Imaging (MBI), Monash University, Clayton, Victoria, 3180, Australia; 6 Life Sciences Computation Centre, Victorian Life Sciences Computation Initiative (VLSCI), Melbourne, Victoria, Australia; 7 Brain Repair and Imaging in Neural Systems (B.R.A.I.N.S) Unit, Department of Experimental Medical Science, Lund University, Lund, Sweden; 8 Lund University Bioimaging Center, Lund, Sweden; University of Ulm, GERMANY

## Abstract

Huntington disease (HD) is a fatal neurodegenerative disorder caused by an expanded CAG repeat in the *huntingtin* gene. Non-motor symptoms and signs such as psychiatric disturbances, sleep problems and metabolic dysfunction are part of the disease manifestation. These aspects may relate to changes in the hypothalamus, an area of the brain involved in the regulation of emotion, sleep and metabolism. Neuropathological and imaging studies using both voxel-based morphometry (VBM) of magnetic resonance imaging (MRI) as well as positron emission tomography (PET) have demonstrated pathological changes in the hypothalamic region during early stages in symptomatic HD. In this investigation, we aimed to establish a robust method for measurements of the hypothalamic volume in MRI in order to determine whether the hypothalamic dysfunction in HD is associated with the volume of this region. Using T1-weighted imaging, we describe a reproducible delineation procedure to estimate the hypothalamic volume which was based on the same landmarks used in histologically processed postmortem hypothalamic tissue. Participants included 36 prodromal HD (pre-HD), 33 symptomatic HD (symp-HD) and 33 control participants who underwent MRI scanning at baseline and 18 months follow-up as part of the IMAGE-HD study. We found no evidence of cross-sectional or longitudinal changes between groups in hypothalamic volume. Our results suggest that hypothalamic pathology in HD is not associated with volume changes.

## Introduction

Huntington’s disease (HD) is a fully penetrant autosomal dominant neurodegenerative disorder caused by an expansion of a CAG repeat in the *huntingtin* gene [1]. HD has traditionally been considered a movement disorder, however, non-motor symptoms and signs such as psychiatric symptoms, cognitive dysfunction, disrupted circadian rhythm, as well as metabolic alterations often manifest [2,3,4,5,6,7]. The hypothalamus plays a critical role in the regulation of sleep, emotion and appetite [8,9,10]. The very few neuropathological studies investigating the hypothalamus in HD have demonstrated loss of neuropeptides involved in the regulation of sleep, emotion and appetite, suggesting that hypothalamic dysfunction may be involved in the development of several non-motor related features [11,12,13,14,15,16]. Studies with positron emission tomography (PET) have shown reductions in dopamine D2 receptor levels as well as microglia activation in the hypothalamic region during the prodromal stage of HD [17,18].

Studies using voxel based morphometry (VBM) analyses of magnetic resonance imaging (MRI) scans have indicated that there is a reduction in signal intensity in voxels in the hypothalamic region in early symptomatic stages of the disease [19,20]. We have previously reported, using both VBM and logistic regression analyses of cross-sectional MRI from the Predict-HD study, that hypothalamic changes are present up to 15 years before the predicted onset of motor symptoms [21]. A number of pathological findings have also been made in the hypothalamus of several animal models of HD [10]. Interestingly, the development of non-motor features such as metabolic dysfunction and depressive-like behavior can be prevented with the hypothalamic inactivation of the mutant *huntingtin* gene in the BACHD mouse model of HD [22,23]. Hence, the hypothalamus has emerged as an important site of pathology in HD that should be considered when developing therapeutic strategies to alleviate non-motor aspects of the disease.

Structural MRI of the striatum and the cerebral cortex has repeatedly revealed significant reductions in volume during prodromal HD (pre-HD) compared to controls [24,25,26,27,28,29,30]. Although functionally impaired, the extent to which hypothalamic volume is affected in HD is not fully known. This is because the technique to delineate the hypothalamus in MRI is not well established, likely due to difficulties in determining the anatomical borders of the hypothalamus. Nevertheless, a decrease in the hypothalamic volume using structural MRI has been shown in studies of individuals with schizophrenia, affective disorders, and in behavioral-variant frontotemporal dementia [31,32,33,34,35,36,37]. However, the variability of the estimated size of the hypothalamic volumes between studies is high, probably due to different methodological approaches, including discrepancies in defining the hypothalamic region.

The previously reported dysfunction of the hypothalamus in HD does not indicate if it is associated with changes of the hypothalamic volume per se [11,12,13,14,15,16,38]. In this study, our aim was to establish a robust method to measure the volume of the hypothalamus, and then to apply this method to determine whether hypothalamic volume is altered in individuals with pre-HD and symptomatic HD (symp-HD). Knowledge about the volume of the hypothalamus in HD is important for design of imaging studies investigating functional changes of this region as well as for the application of potential therapeutic interventions targeting this area. We have previously used histologically-processed postmortem human hypothalamic tissue to establish a reproducible method to delineate the hypothalamic region using stereological principles [13]. We have since refined this method to measure the hypothalamic volume *in vivo* in HD (via T1-weighted imaging). We used data from IMAGE-HD, a longitudinal multi-modal neuroimaging study based in Melbourne [29,30,39,40,41,42,43]. Because Image-HD used 3T MRI, this also provided higher resolution data than were available from our previous studies, which used 1.5 T MRI [10,21]. Using our new delineation method, we compared hypothalamic volume between individuals with pre-HD, symp-HD and healthy controls at baseline and at 18 months follow-up.

## Material and Methods

### Ethics Statement

The study was approved by the Monash University and Melbourne Health Human Research Ethics Committees, and each participant gave written informed consent.

### Participants

The IMAGE-HD study is a longitudinal multimodal neuroimaging study including clinical, neurocognitive and neuropsychiatric assessments of 36 pre-HD, 36 symp-HD and 36 healthy controls [29,30,39,40,41,42,43]. For the current investigation, we included data from 36 pre-HD, 34 symp-HD and 33 healthy controls collected as part of the IMAGE-HD study. For this analysis, images from 2 symp-HD and 3 healthy control cases were excluded from the original dataset as there were movement artifacts that compromised the image quality.

Demographic and clinical data collected at baseline and at an 18 months follow-up are presented in Tables 1 and 2. Pre-HD and symp-HD participants underwent genetic testing and had a CAG repeat length ranging from 39 to 50. All participants were clinically assessed using Unified Huntington's Disease Rating Scale (UHDRS) motor subscale. HD participants with a UHDRS motor score ≤5 were included in the pre-HD group and those with UHDRS motor score >5 were included in the symp-HD group. Estimated years-to-onset of diagnostic motor symptoms were calculated for the pre-HD participants using the formula established by Langbehn and colleagues, accounting for CAG repeat length and current age [44]. Healthy controls were matched for age, gender and IQ (National Adult Reading Test 2^nd^ edition, NART-2) [45] to the pre-HD individuals.

**Table 1:** Demographic, cognitive and neuropsychiatric data of the Image-HD study participants at baseline.

	Control	Pre-HD	Symp-HD
n	33	36	33
Gender (F/M)	22/11	22/14	14/19
Age (years)	44 ± 13	42 ± 10	53 ± 9
CAG repeat length		42 ± 2	43 ± 2
UHDRS—motor subscale		0.86 ± 1.22	18.30 ± 10.68
Estimated years to onset		15 ± 7	
Disease burden score		270 ± 53	376 ± 72
Duration of illness (years)			2 ± 2
Verbal IQ	118 ± 10	116 ± 11	115 ± 11
SDMT	56 ± 11	52 ± 9	36 ±12
Stroop	110 ± 17	105 ± 17	84 ± 22
SCOPI – total OCD	80 ± 20	82 ± 25	90 ± 25
FrSbe – total score	87 ± 26	91 ± 23	92 ± 23
HADS: Anxiety	5 ± 3	7 ± 4	5 ± 4
HADS: Depression	2 ± 3	3 ± 3	3 ± 2
BDI II	3 ± 3	9 ± 10	8 ± 7

Data is presented as mean ± SD. UHDRS-M: Unified Huntington’s Disease Rating Scale—motor subscale score (Pre-HD, UHDRS<5; Symp-HD, UHDRS≥5); CAG: number of repeats > 40 is full penetrance; SDMT: Symbol Digit Modalities Test; Stroop: Stroop speeded word reading task (number of correct words); FrSBe: Frontal Systems Behaviour Scale; SCOPI: Schedule of Compulsions Obsessions and Pathological Impulses; HADS A: Hospital Anxiety and Depression scale—anxiety sub score; HADS D: Hospital Anxiety and Depression scale – depression sub score; BDI II: Beck Depression Inventory Version II score.

**Table 2:** Demographic, cognitive and neuropsychiatric data of the Image-HD participants at 18 months follow-up.

	Control	Pre-HD	Symp-HD
n	25	30	27
Gender (F/M)	17/8	18/12	10/17
Age (years)	44 ± 12	42 ± 10	54 ± 9
CAG repeat length		42 ± 2	43 ± 2
UHDRS-motor subscale		3 ± 4	21 ± 12
Estimated years to onset		14 ± 7	
Disease burden score		281 ± 56	382 ± 74
Duration of illness (years)			4 ± 2
Verbal IQ	120 ± 10	116 ± 11	116 ± 12
SDMT	57 ± 7	52 ± 10	37 ± 11
STROOP	104 ± 15	101 ± 14	83 ± 18
SCOPI – total OCD	74 ± 14	75 ± 18	84 ± 23
FrSbe – total score	75 ± 17	89 ± 22	90 ± 21
HADS: Anxiety	4 ± 2	6 ± 5	4 ± 4
HADS: Depression	2 ± 2	3 ± 4	3 ± 3
BDI II	3 ± 3	7 ± 8	7 ± 6

Data is presented as mean ± SD. UHDRS-M: Unified Huntington’s Disease Rating Scale—motor subscale score (Pre-HD, UHDRS<5; Symp-HD, UHDRS≥5); CAG: number of repeats > 40 is full penetrance; SDMT: Symbol Digit Modalities Test; Stroop: Stroop speeded word reading task (number of correct words); FrSBe: Frontal Systems Behaviour Scale; SCOPI: Schedule of Compulsions Obsessions and Pathological Impulses; HADS A: Hospital Anxiety and Depression scale—anxiety sub score; HADS D: Hospital Anxiety and Depression scale – depression sub score; BDI II: Beck Depression Inventory Version II score.

A battery of neurocognitive tests was performed at both time points. The tests assessed visuomotor speed and attention (Symbol Digit Modalities Test, SDMT) [46] and speeded reading (Stroop word test) [47]. Participants were also assessed for behaviours associated with frontal-striatal brain dysfunction (Frontal system Behaviour Scale, FrSBe), and psychiatric disturbances (Schedule of Obsessions, Compulsions and Psychological Impulses, SCOPI; [48]; Hospital Anxiety and Depression Scale, HADS; [49]; Beck Depression Inventory Version II, BDI-II) (see Tables 1 and 2).

### MRI Acquisition

MRI images were obtained on a Siemens 3 Tesla scanner. T1-weighted images were acquired for each participant using the following acquisition sequence parameters: 192 slices, 0.9 mm slice thickness, 0.8 mm x 0.8 mm in-plane resolution, TE = 2.59 ms, TR = 1900 ms, flip angle = 9°.

### Image analysis

Processing and all manual measurements of the images after acquisition were performed with the ANALYZE 10.0 software package (Biomedical Imaging Resource, Mayo foundation, Rochester, MN) with a digitized pen and drawing pad. The volumetric measurements of the hypothalamus were derived on T1-weighted images, which were preprocessed by acquiring cubic spine interpolation and a resizing of voxels to 0.42 x 0.42 x 0.45 mm^3^. The original slices were reformatted into the coronal plane.

### Segmentation of the hypothalamus

**Fig 1:**
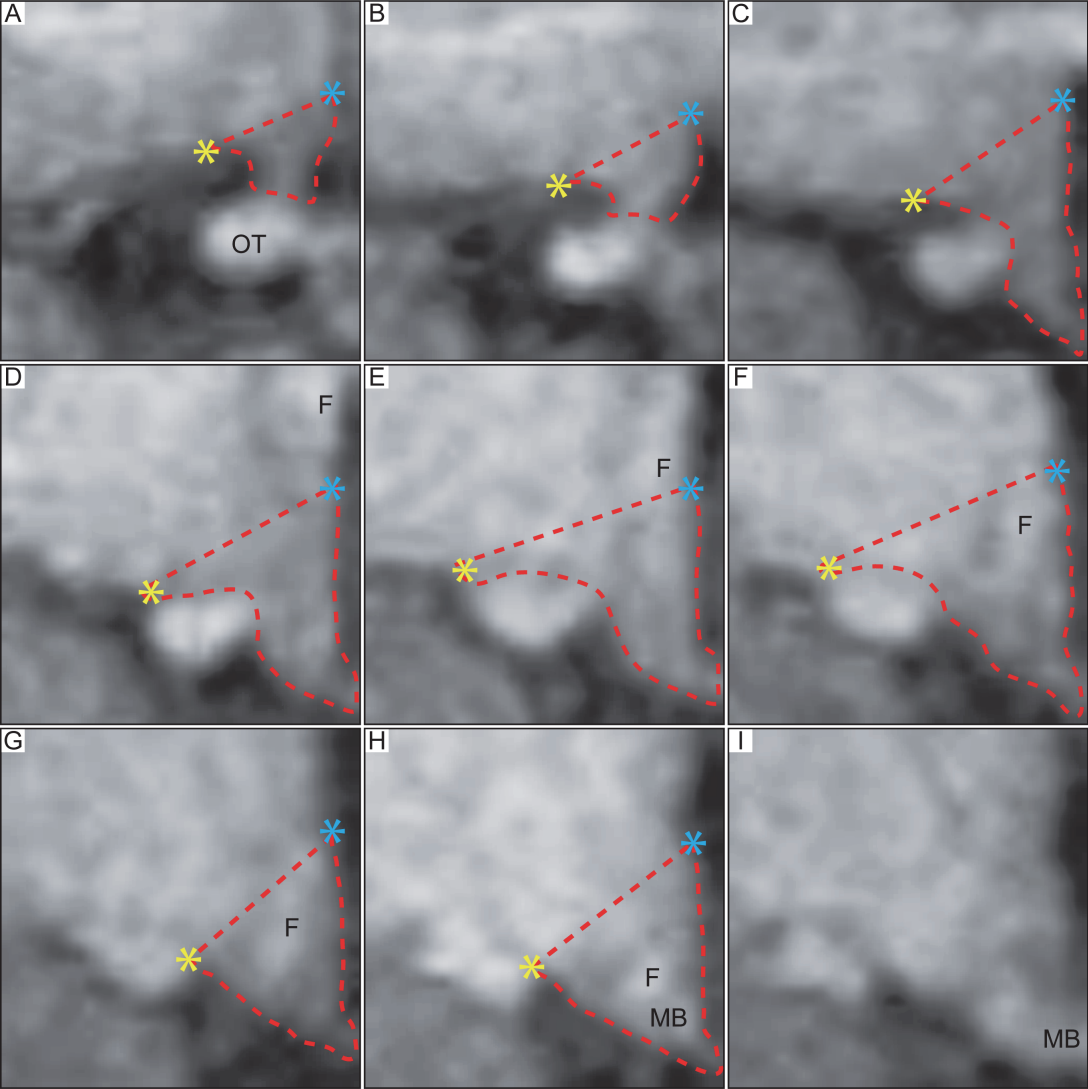
Delineation of the hypothalamic region in 3T MR images based on postmortem hypothalamic material. Overview of the boundaries to delineate the hypothalamus in postmortem human tissue applied to T1-weighted MRI acquired at 3T [13]. A-I represents the hypothalamic region in a coronal plane, from rostral to caudal direction. The red dashed lines illustrate how the hypothalamic region was delineated. Landmarks such as the hypothalamic sulcus (represented by a blue star) and the lateral or medial edge of the optical tract (represented by yellow star) were identified for the delineation and a straight line between these two points was drawn to set the superior/lateral border of the area. The optical tract was excluded in all slides. Abbreviations: optical tract, OT; fornix, F; mammillary body, MB.

Manual segmentation of hypothalamus in T1 weighted images was carried out by two independent raters on a subset of cases in order to establish the boundaries that would yield a reproducible estimation of the volume. The delineation of the hypothalamus was first made by applying the landmarks and boundaries described in the postmortem material on the MRI (Fig. 1; [13]). To establish a robust and reproducible method to estimate the hypothalamic volume using T1-weighted 3T MRI scans, we began by using the landmarks previously employed in coronal sections of fixed postmortem hypothalamic tissue processed with Nissl staining [13]. Briefly, the first slide to be included in the anterior direction was where the preoptic hypothalamus was present and where the optic chiasm was first seen attached to the ventral part of the septal area in a coronal plane at bregma level −1.3 mm (according to the Atlas of the Human Brain by Mai et al [50] (Fig. 1 A-I). A superior border was drawn as a straight line from the hypothalamic sulcus to the most lateral point of the optic tract throughout the majority of the hypothalamic region until it was shifted to being drawn medial to the optical tract. This shift was guided by the medial presence of limiting medullary lamina of globus pallidus in the Nissl-stained sections at the bregma level of 6.7 mm but was less apparent in the MRI. The medial border of the hypothalamus was drawn by following the 3^rd^ ventricle. The inferior border was set at the junction to the optical chiasm for the anterior sections and then defined by the border to cerebrospinal fluid in more posterior sections. The final section was included at the level when the fornix appears to be merged with the mammillary nucleus at bregma level 9.3 mm. The optical tract was excluded in all slides.

To ensure high intra-rater reliability, all raters initially repeated each estimation of the hypothalamic volume in 15 randomly selected cases three times. The coefficient of variation (CV) was then calculated for each case to assess the accuracy and reproducibility according to the following formula: CV = (standard deviation of the hypothalamic or ventricle volume) /mean of the hypothalamic or ventricle volume) x 100. A CV value <4% was considered acceptable. Once this was achieved, raters began to perform the hypothalamic volume measurements reported in this study.

**Fig 2:**
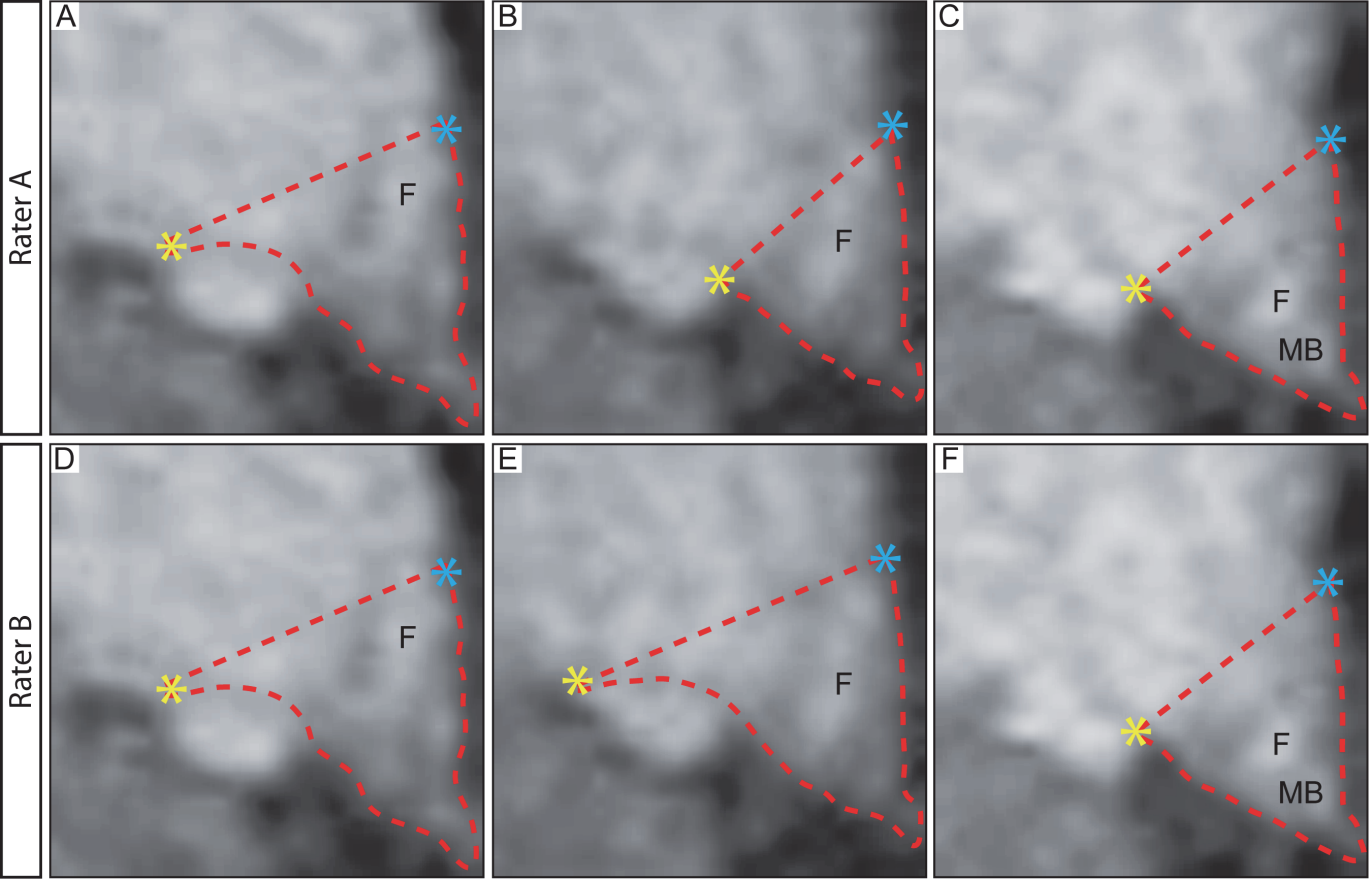
Discrepancy between raters when applying delineation boundaries from postmortem material to MRI. The application of the landmarks from postmortem material to MRI resulted in discrepancy between the results obtained from the two independent raters. The difference of the mean for the hypothalamic volume in the assessed control groups was 15 ± 11% (mean ± SD) between the two raters and the intraclass correlation coefficient (ICC) between the two raters was 0.562. Upon inspection of the delineation made by the two raters, the major discrepancy was found to be when the decision was made to shift the superior/lateral border from being lateral to medial of the optical tract (A-H). Abbreviations: fornix, F; mammillary body, MB.

**Fig 3:**
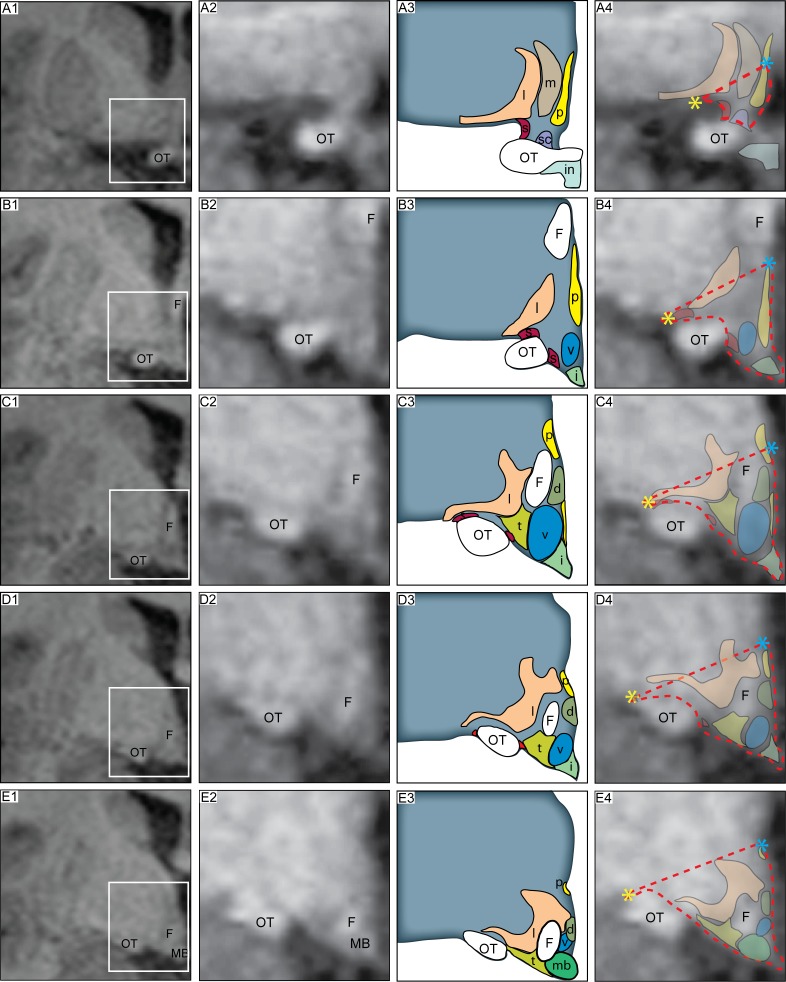
Reproducible delineation of the hypothalamic region in 3T MR images. Overview of the modified boundaries to delineate the hypothalamus in T1-weighted MRI acquired at 3T that yielded high reproducibility between raters (difference of the mean for the hypothalamic volume between the two raters: 2 ± 6%, ICC between the two raters = 0.937). From a rostral to caudal direction, A1-E1 column is a representative 3T MR image in a coronal plane, where the white box indicates the region of interest. Column A2-E2 represents a close up of the hypothalamic region. A schematic overview of the corresponding level adapted from Mai et al., human brain atlas [50] indicated in column A3-E3. Column A4-E4 illustrates an overlap of the schematic overview on the MR image. The red dashed lines illustrate how the hypothalamic region was delineated. Landmarks such as the hypothalamic sulcus (represented by a blue star) and the lateral edge of the optical tract (represented by yellow star) were identified for the delineation and a straight line between these two points was drawn to set the superior/lateral border of the area. The optical tract was excluded in all slides. Abbreviations: dorsomedial hypothalamic nucleus, d; fornix, F; infundibular stalk, i; lateral hypothalamus, l; mammillary body, MB; medial preoptic nucleus, m; optical tract, OT; paraventricular nucleus, P; suprachiasmatic nucleus, sc; supraoptic nucleus, s; ventromedial hypothalamic nucleus, v; tuberomammillary hypothalamic nucleus, t.

Due to the discrepancy in results between the raters (described in the Results section and illustrated in Fig. 2), the superior/lateral border was modified (described in the Results section and illustrated in Fig. 3). A single rater then estimated the hypothalamic volume bilaterally on all cases using between 15 to 20 sequential slices from a rostral to caudal direction. The raters were blinded to all clinical data.

### Measurement of the total intracranial volume

The total intracranial volume (ICV) was calculated for each participant to allow adjustments of the hypothalamic volume between subjects with different head sizes. First all non-brain tissue was removed from the T1 weighted images of each participant with BET (Brain Extraction Tool, FMRIB’s Software Library FSL, 4.1.6) [51]. BET did not work satisfactorily in T1 images from 5 participants (due to advanced atrophy). In these cases, the best output of BET was manually corrected to achieve adequate extraction of the brain. Secondly, FAST (FSL’s Automated Segmentation Tool) was used to segment each participant’s T1 weighted image, whilst correcting for spatial intensity variations, into tissue type: grey matter (GM), white matter (WM) and (ventricular and intergyral) cerebrospinal fluid (CSF). The ICV was then estimated from these segmentations.

### Statistical analyses

Statistical analyses were performed using the SPSS 19 statistical package (SPSS inc. Chicago, IL, USA). Statistical differences were considered significant at p<0.05. Student’s *T*-test, one and three-way analyses of variance (ANOVA) when appropriate were used for group comparisons and controlling for covariates whenever appropriate. For correlations analysis, the Spearman’s Rho correlations were performed between clinical results and volumetric measures.

## Results

### Establishment of a method for delineating hypothalamic volumes

Two independent raters first used the landmarks established in the histologically-processed postmortem tissue to delineate the hypothalamus on MRI from the 33 control cases at baseline. However, applying these landmarks in MRI in the present study imposed discrepancy between the results obtained from the two raters. The mean difference of the hypothalamic volume in the assessed control group was 15 ± 11% (mean ± SD) between the two raters. The intraclass correlation coefficient (ICC) between the two raters was 0.562, indicating that the employed landmarks were not useful for obtaining robust and reproducible measurements. The discrepancy between the raters delineations was mainly due to the decision to shift the lateral border of the hypothalamus from being lateral to medial of the optical tract in the more posterior sections (illustrated in Fig. 2 A-F).

For the next step, we decided to keep the delineation lateral to the optical tract in all sections throughout the hypothalamus in order to reduce this variability, with the awareness that a larger area had to be included (Fig. 3A-I). Two independent raters then estimated the hypothalamic volume in a subset of 25 control cases at baseline based on these new boundaries. We now found that the mean difference of the hypothalamic volume in the control groups improved to 2 ± 6% (mean ± SD) between the two raters. The ICC between the two raters was also high, ICC = 0.937, indicating that these borders provided a reproducible method to estimate the hypothalamic volume in 3T MRI.

### Cross-sectional hypothalamic volumes

**Fig 4:**
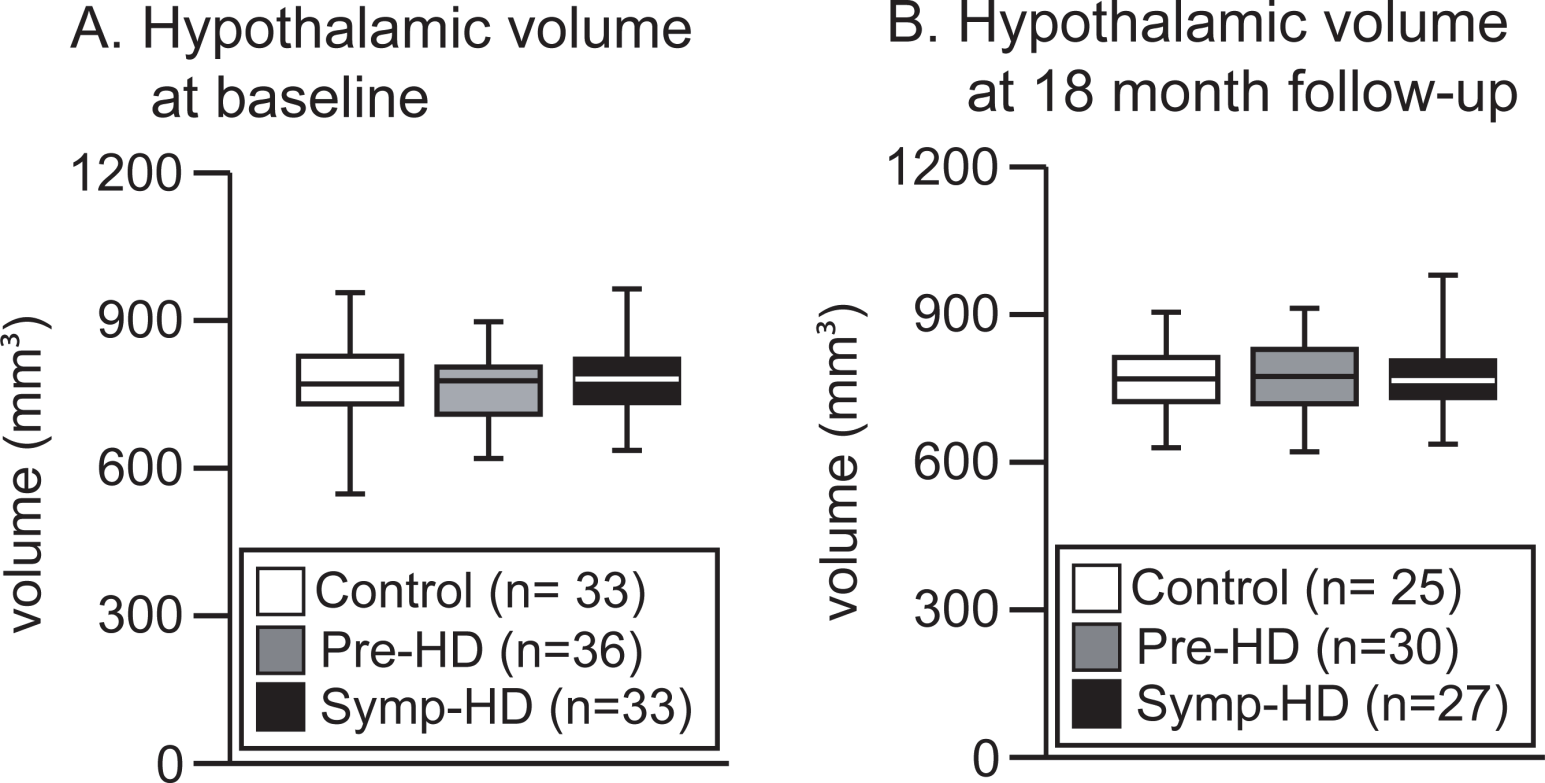
Estimation of the hypothalamic volume in the IMAGE-HD cohort. Boxplots illustrating the estimated hypothalamic volumes in control, pre-HD and symp-HD groups at baseline (A) and the 18 months follow- up time point (B). No statistical changes were observed between the groups at baseline (one-way ANOVA: group (F(2,99) = 0.484, p = 0.618) or at the 18 months follow-up time point (one-way ANOVA: group (F_(2,79)_ = 0.067, p = 0.936).

We then sought to investigate whether the previously reported hypothalamic pathology in HD was associated with changes in hypothalamic volume in individuals with the HD gene. We applied our established landmarks to delineate the hypothalamic volume bilaterally in baseline and 18 month follow-up data from Image-HD. At baseline, the estimated hypothalamic volume was 796± 76 mm^3^ (mean± SD) in the pre-HD group, 757± 83 mm^3^ in the symp-HD group and 770± 28 mm^3^ in the control group (Fig. 4A). A one–factor ANOVA revealed no statistical differences between groups independent of whether the data was analyzed with or without separation by sex. There were also no significant hypothalamic differences for the data obtained at 18-months follow-up (Fig. 4B).

### 18 months longitudinal hypothalamic volumes

**Fig 5:**
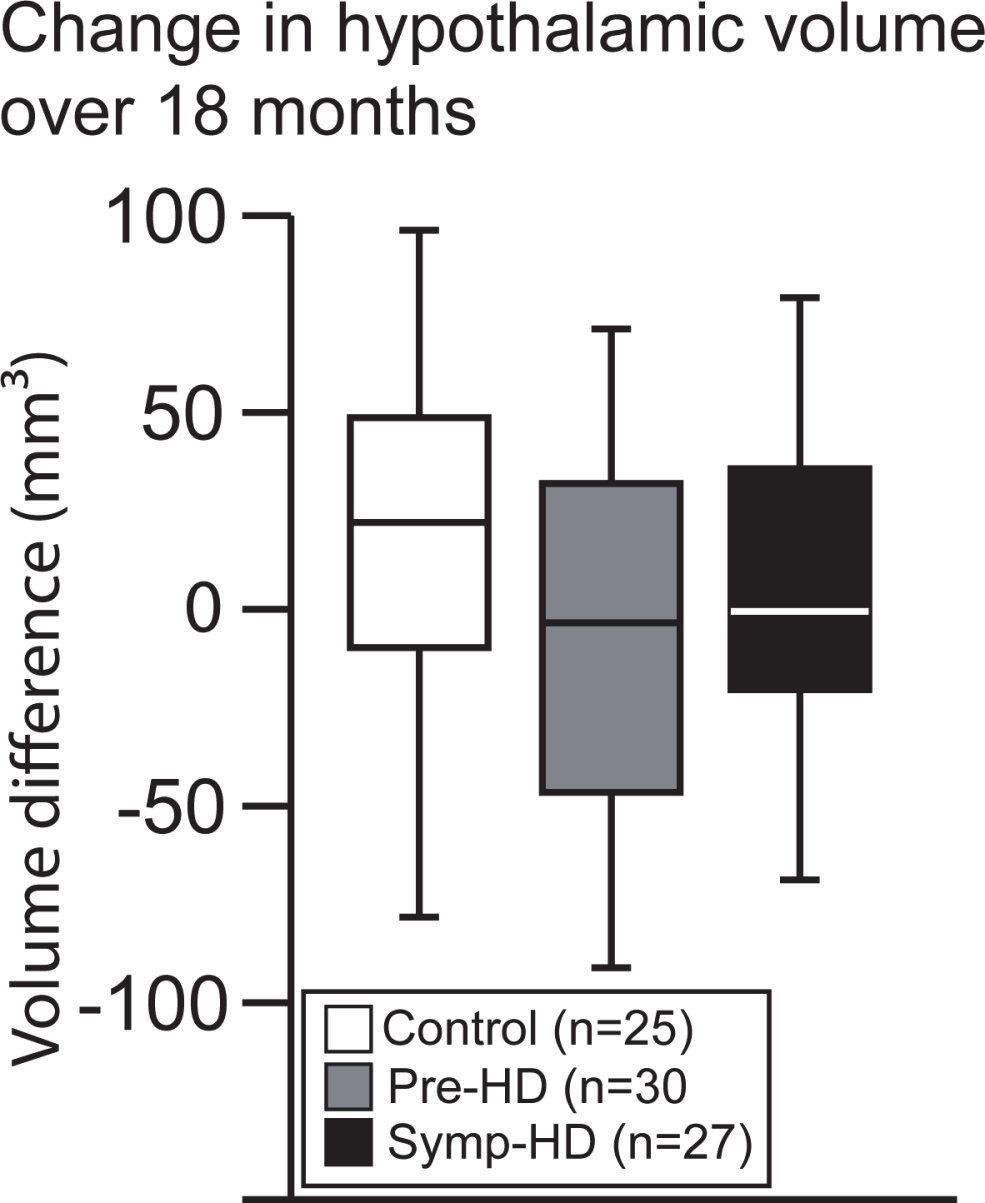
No changes in hypothalamic volume over 18 months. Boxplots illustrating hypothalamic volume differences between the two time points for the three groups. A paired sample t-test revealed no significant changes for the hypothalamic volume between the two time points across the groups; control baseline (mean = 783 mm^3^, SD = 71) and control 18 months (mean = 769 mm^3^, SD = 65), n.s (t_(24)_ = 0.698, p = 0.492), pre-HD baseline (mean = 763 mm^3^, SD = 71) and pre-HD 18 months (mean = 774 mm^3^, SD = 74), n.s (t_(29)_ = −0.541, p = 0.593) and symp-HD baseline (mean = 790 mm^3^, SD = 85) and symp-HD 18 months (mean = 776 mm^3^, SD = 80), n.s (t_(26)_ = 0.574, p = 0.571).

We then investigated whether there would be a change in the individual hypothalamic volume over an 18 months period. Pair-wise t-test analysis revealed no significant longitudinal differences in the hypothalamic volume across any of the groups (Fig. 5).

### No differences in hypothalamic volumes across groups in the Image-HD cohort

**Fig 6:**
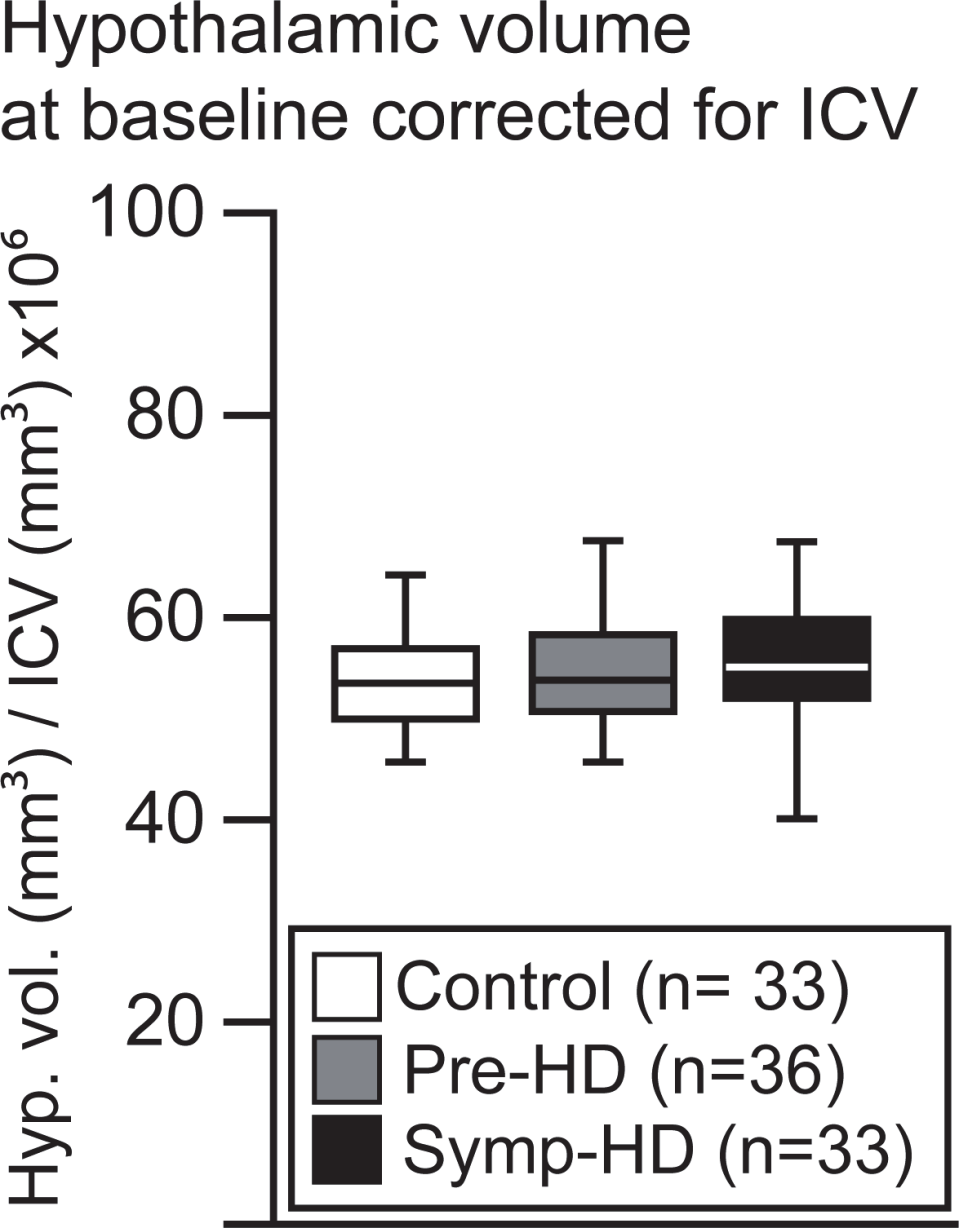
Hypothalamic volumes corrected for ICV. Boxplots illustrate hypothalamic volumes when corrected for ICV for the control, pre-HD and symp-HD groups at baseline. A four-way ANOVA revealed only a significant effect of ICV (ICV: F_(1,96)_ = 16.826, p = 0.001) but not for the other parameters (group: n.s (F_(2,96)_ = 0.597, p = 0.553), sex: F_(1,96)_ = n.s (F = 3.804, p = 0.054), age: n.s. (F_(1,96)_ = 0.165, p = 0.685)). Once adjusted for ICV, a three-way ANOVA was performed to assess the effect of HD gene status, sex and age on the hypothalamic volume estimates. No significant effects were found (group: n.s (F_(2,92)_ = 0.838, p = 0.436), sex: F_(1,92)_ = n.s (F = 0.927, p = 0.338), and age: n.s. (F_(1,92)_ = 0.576, p = 0.564)).

Hypothalamic volume has been previously shown to be influenced by demographic parameters, such as age and sex [33,52,53]. Moreover, hypothalamic volume measurements have also been corrected for ICV in several reports [33,36]. We performed an ANOVA to assess hypothalamic volume differences between the three groups with sex, ICV and age as covariates. The statistical analysis demonstrated a significant effect of ICV on the hypothalamic volume estimates (F_1,96_ = 16.826, p<0.0001). We then adjusted the hypothalamic volume for ICV at baseline (Fig. 6) and performed a three factor ANOVA to assess whether there was an effect of HD gene status, sex and age on the ICV-corrected hypothalamic volume estimates. The statistical analysis revealed no significant effect of either factors on the hypothalamic volumes, indicating that there was no significant differences in the hypothalamic volumes between HD gene carriers and controls.

### Correlation between ICV-adjusted hypothalamic volumes and clinical parameters

Finally, we sought to determine whether any relationships exist between size of hypothalamic volume and various clinical parameters such as UHDRS, disease burden score, duration of illness, neurocognitive parameters; SDMT and STROOP, as well as neuropsychiatric parameters; FrSbe, SCOPI, HADS-A AND HADS-B and BDI II. Correlation analyses were performed for the ICV-adjusted hypothalamic volumes and the clinical, neurocognitive, and neuropsychiatric data collected from all the participants reported in the methods section above (and only at baseline). We found a significant positive correlation between disease duration and ICV-adjusted hypothalamic volume for the symp-HD group (Spearman’s Rho = 0.489, p < 0.004; Table 3). There were no other significant correlations for any other measure.

**Table 3:** Results from non-parametric correlation analyses for ICV-corrected hypothalamic volumes and selected clinical cognitive and neuropsychiatric data collected for all the participants in the IMAGE-HD cohort at baseline.

	Spearman's Rho	p-value
UHDRS	0.234	0.053
Diagnostic score	0.219	0.071
Disease burden score	0.173	0.155
Years to onset	−0.198	0.253
Duration of illness	0.489	0.004
Verbal IQ	−0.057	0.571
SDMT	−0.112	0.262
Stroop	−0.074	0.458
SCOPI	−0.112	0.264
FRSBE	0.022	0.823
HADS A	0.060	0.547
HADS D	0.132	0.187
BDI II	0.085	0.187

UHDRS-M: Unified Huntington’s Disease Rating Scale—motor subscale score (Pre-HD, UHDRS<5; Symp-HD, UHDRS≥5)SDMT: Symbol Digit Modalities Test; STROOP: STROOP speeded word reading task (number of correct words); FrSBe: Frontal Systems Behaviour Scale; SCOPI: Schedule of Compulsions Obsessions and Pathological Impulses; HADS A: Hospital Anxiety and Depression scale—anxiety sub score; HADS D: Hospital Anxiety and Depression scale – depression sub score; BDI II: Beck Depression Inventory score Version II. Univariate associations between two continuous variables were analyzed using Spearman’s correlation, p-values < 0.05 were considered significant.

## Discussion

The hallmark pathology of HD is comprised of atrophy and cell death of the striatum of the basal ganglia [54]. Volumetric analysis of the striatum is therefore a useful and established tool to determine pathology and disease progression in HD in both postmortem brain sections and in MRI [26,27,28,29,30,54,55,56,57,58,59,60,61]. Less is known about to what extent hypothalamic volume is affected in HD, however. Neuropathological changes in the hypothalamus in HD have been reported previously using a number of different techniques [10]. Loss of the neuropeptide orexin (hypocretin) expressed in the lateral hypothalamus has been repeatedly described [11,12,13]. Other studies have detected loss of the neuropeptides vasopressin, oxytocin, vasoactive intestinal polypeptide, somatostatin and neuropeptide Y as well as increased numbers of neurons expressing cocaine and amphetamine regulated transcript in different hypothalamic nuclei [13,15,38,62]. We have previously performed a volumetric analysis of the hypothalamic region in Nissl/myelin stained brain sections using stereological principles in a small cohort of HD and control cases [13]. In the study by Gabery et al. (2010) [13], we estimated the unilateral hypothalamic volume to be 363 ± 56 mm^3^ in HD cases of Vonsattel grades II-III and 406 ± 49 mm^3^ in control cases. The 11% difference in volume was not statistically significant in that cohort of cases (n = 8–9/group). In the present study, we used a similar albeit a slightly modified delineation approach to estimate the hypothalamic volume *in vivo*. However, we did not detect any significant cross-sectional or longitudinal differences in hypothalamic volume between pre-HD, symp-HD or healthy controls. Data from the IMAGE-HD study therefore did not yield detectable hypothalamic volumetric changes in the cohort size investigated in the present study. Hence, it appears that the hypothalamic alterations reported previously are not associated with changes of the hypothalamic volume.

There may be several explanations to why there is no atrophy of the hypothalamic region in HD despite the reported hypothalamic dysfunction. From post-mortem analysis of HD brains, we know that there is a selective but partial degeneration of certain neuronal populations such as the ones expressing orexin, oxytocin or vasopressin which collectively represent about 5% of the neurons in this region, and that other cell populations are unaffected [13]. Effects on small cell populations in specific nuclei of the hypothalamus may not be apparent when the whole region is assessed. In fact, previous studies have reported around 30% loss of the number of orexin-immunopositive neurons as well as 23% loss of neurons in the paraventricular nucleus (PVN) in the hypothalamus in HD although stereological estimation of the total number of hypothalamic neurons did not indicate any differences in cell numbers between HD and control cases [11,12,13]. In a previous postmortem study, we did not find any volume changes of the PVN despite the loss of 23% of neurons in that nucleus as assessed using stereological principles of Nissl-stained material (Gabery et al., 2010). Finally, reduction in immunopositive cells and reduced expression of neuropeptides may be a sign of neuronal dysfunction rather than neuronal loss, and hence would not be expected to lead to any changes in volume of a brain region. However, in the present study, we found a statistically significant positive correlation between hypothalamic volume and disease duration, indicating that the volume of the hypothalamus may be larger in more advanced disease. This may be due to inflammation as increased microglia activation has been found in the hypothalamus in HD [17]. Such changes would be interesting to explore further in a cohort of more advanced stages of HD.

Previous studies have used various methods to delineate the hypothalamus in MRI or histologically processed postmortem material (Table 4). However, there has been high variability amongst these reports regarding size of the hypothalamic volume *in vivo* (ranging between 360–1050 mm^3^) due to the lack of an established method to perform these measurements. In this study, we refined our previous anatomical landmarks set on histologically-processed post mortem tissue, and applied these on T1 weighted 3T MR images for a reproducible segmentation of the hypothalamus. By using these landmarks, a high degree of inter-rater reliability was achieved (ICC = 0.937). The hypothalamic volume reported herein is comparable to our previous study, in which we performed measurements using Nissl-myelin staining on fixed post mortem hypothalamic tissue (Table 4) [13]. Our volume estimation results also compare to a previous study based on 3T MRI [63], however, with our approach we achieved a higher inter-rater ICC, indicating higher reproducibility by applying our method. Nevertheless, by applying different methods, altered hypothalamic volume has been reported for diseases such as Alzheimer's disease [64,65], frontotemporal dementia [37], schizophrenia (including schizophrenia [33], alcoholism [66,67] and anxiety disorders [33]. It is important to establish to what extent such changes can be reproduced in different cohorts and to perform studies using a similar delineation method across the diseases. Hence, the method presented in this study will be useful for estimating the hypothalamic volume in a number of conditions with hypothalamic pathology.

**Table 4:** Overview of reported estimations of the hypothalamic volume in clinical studies.

Study	Volumetric approach	Delineation procedure	Region of interest	Control cohort	Age (years, mean ± SD)	Volume mm^3^ (mean ± SD)	Software
Callen et al., 2001[65]	1.5T MRI	n.a	Unilateral hypothalamus (MB excluded)	40	n.a	300 ± 40	n.a
Peper et at., 2005 [68]	1.5T MRI	Manual	Bilateral hypothalamus (MB included)	female: 46, male: 39	Female: 12 ± 1 Male: 11.6 ± 1	Female: 1010 ± 90 Male: 1050 ± 120	n.a
Bielau et al., 2005[69]	Post mortem, stereology	Manual	Bilateral hypothalamus (MB included)	22	49.9 ± 11.6	1410 ± 302	n.a
Hulshoff Pol et al., 2006[35]	1.5T MRI	Manual	Bilateral hypothalamus (MB excluded)	Female: 6 Male: 9	Female: 23 ± 6 Male: 25 ± 8	Female: 1000 ± 50 Male: 1050 ± 180	n.a
Goldstein et al., 2007[33]	1.5T MRI	Semi-automated	Bilateral hypothalamus (MB included)	Female: 21 Male: 27	40.5 ± 10.8	Female: 780 ± 160 Male: 920 ± 110	n.a
Koolschijn et al., 2008 [36]	1.5T MRI	Manual	Bilateral hypothalamus (MB excluded)	44	n.a	1040 ± 100, 1010 ± 140, 970 ± 130, 1040 ± 130	n.a
Bogerts, 2010 [70]	Post mortem, stereology	Manual	Bilateral hypothalamus (MB included)	23	50 ± 12	Female: 664.9 ± 101.6 Male: 765.9 ± 102.3	n.a
Gabery et al., 2010 [13]	Post mortem, stereology	Manual	Unilateral hypothalamus (MB excluded)	9	61 ± 5	406 ± 49	VIS software (Visiopharm, Horsholm, Denmark)
Piguet et al., 2011 [37]	Post mortem, stereology	Manual	Unilateral hypothalamus (MB excluded)	6	71.6 ± 5.9	Anterior hypothalamus: 156 ± 39 Posterior hypothalamus: 193 ± k43	Stereo Investigator 8.0 (MBF Bioscience, Microbrightfield Inc., USA)
Klomp et al., 2012 [32]	1.5T MRI	Manual	Bilateral hypothalamus (MB excluded)	156	n.a	1039 ± 139	n.a
Tognin et al., 2012 [63]	3T MRI	Manual	Bilateral hypothalamus (MB excluded)	Female: 9 Male: 17	32.23 ± 3.81	Right hypothalamus: 360 ± 50 Left hypothalamus: 360 ± 40	BRAINS2 software
Markis et al., 2013 [71]	1.5T MRI	Semi-automated	Bilateral hypothalamus (MB included)	Female: 18 Male: 26	Females: 38 ± 9.6 Male: 42 ± 11.5	Females: 790 ± 140 Male: 910 ± 110	n.a
Ha et al., 2013 [72]	1.5T MRI	Manual	Bilateral hypothalamus (MB included)	Lean individuals: 29 Obese individual: 62	Lean individuals: 17.3 ± 1.6 Obese individual: 17.5 ± 1.8	Lean individuals: 1480 ± 120 Obese individual: 1540 ± 220	MIDAS.1.11
Terlevic et al., 2013 [73]	1.5T MRI	Manual	Bilateral hypothalamus (MB excluded)	Female: 14, Male: 7	36± 14	Right hypothalamus: 360 ± 40 Left hypothalamus: 340 ± 30	BRAINS2 software
Bielau et al., 2013 [69]	Post mortem, stereology	Manual	Bilateral hypothalamus (MB included)	23	50.17 ± 11.36	Right hypothalamus: 702 ± 164 Left hypothalamus: 701 ± 164	n.a
Schindler et al., 2013 [74]	7T MRI	Semi-automated	Bilateral hypothalamus (MB included)	10	39 ± 14	1130.64 ± 110	ITK-SNAP version 2.14-rcl

Abbreviations MB: mammillary body, T: tesla, MRI: magnetic resonance imaging, n.a: not available

In conclusion, we have developed a reproducible method to estimate the volume of the human hypothalamus using 3T MRI. By using this technique, we could not detect any differences in hypothalamic volume between HD gene carriers and controls from the IMAGE-HD study. Animal studies using the BACHD mouse model of HD have suggested that huntingtin lowering therapies targeting the hypothalamus may have beneficial effects on non-motor features such as metabolic dysfunction and depression [22,23]. Hence, lack of atrophy in the hypothalamic region in *HD* gene carriers, even in the symptomatic stages of the disease, suggests that it would possible to target this area with gene therapy, e.g. using huntingtin lowering RNA knockdown strategies.
